# The Implantation of Bioactive Glass Granules Can Contribute the Load-Bearing Capacity of Bones Weakened by Large Cortical Defects

**DOI:** 10.3390/ma12213481

**Published:** 2019-10-24

**Authors:** Nicole A. P. van Gestel, Floor Gabriels, Jan A. P. Geurts, Dennis J. W. Hulsen, Caroline E. Wyers, Joop P. van de Bergh, Keita Ito, Sandra Hofmann, Jacobus J. Arts, Bert van Rietbergen

**Affiliations:** 1 Orthopaedic Biomechanics, Department of Biomedical Engineering, Eindhoven University of Technology, P.O. Box 513, 5600 MB Eindhoven, The Netherlands; n.a.p.v.gestel@tue.nl (N.A.P.v.G.); d.hulsen@jbz.nl (D.J.W.H.); k.ito@tue.nl (K.I.); j.arts@mumc.nl (J.J.A.); 2 Institute for Complex Molecular Systems, Department of Biomedical Engineering, Eindhoven University of Technology, P.O. Box 513, 5600 MB Eindhoven, The Netherlands; 3 Department of Orthopaedic Surgery, Research School CAPHRI, Maastricht University Medical Centre, PO Box 5800, 6229 HX Maastricht, The Netherlands; j.geurts@mumc.nl; 4 MICT Department, Jeroen Bosch Ziekenhuis, P.O. Box 90153, 5200 ME ’s-Hertogenbosch, The Netherlands; 5 Department of Internal Medicine, VieCuri Medical Center, Tegelseweg 210, 5912 BL Venlo, The Netherlands; cwyers@viecuri.nl (C.E.W.);; 6 Department of Internal Medicine, NUTRIM School for Translational Research in Metabolism, Maastricht UMC+, PO Box 5800, 6229 HX Maastricht, The Netherlands; 7 Biomedical Research Institute, Hasselt University, Agoralaan Building D, 3590 Diepenbeek, Belgium

**Keywords:** osteomyelitis, treatment, bioactive glass granules, stiffness, mechanical properties, finite element analysis, load sharing

## Abstract

Bioactive glass (BAG) granules (S53P4) have shown good clinical results in one-stage treatment of osteomyelitis. During this treatment, a cortical window is created, and infected bone is debrided, which results in large defects that affect the mechanical properties of the bone. This study aimed to evaluate the role of BAG granules in load-bearing bone defect grafting. First, the influence of the geometry of the cortical window on the bone bending stiffness and estimated failure moments was evaluated using micro finite element analysis (µFE). This resulted in significant differences between the variations in width and length. In addition, µFE analysis showed that BAG granules contribute to bearing loads in simulated compression of a tibia with a defect grafted with BAG and a BAG and bone morsel mixture. These mixtures potentially can unload the cortical bone that is weakened by a large defect directly after the operation by up to approximately 25%, but only in case of optimal load transfer through the mixture.

## 1. Introduction

Bioactive glass (BAG) is a promising biomaterial for the regeneration of large bone defects and has been studied since the late 1960s [1,2]. The bone bonding properties of BAGs enable the regeneration of bone in several clinical indications [3,4]. The specific S53P4 composition has shown good results in the treatment of bone infections (osteomyelitis) in granular form, as this composition shows antibacterial effects beside its bone bonding properties [4,5,6,7]. In Europe, over 100 patients suffering from osteomyelitis have successfully been treated with S53P4 BAG granules [8]. The possibility to treat osteomyelitis in this one-stage fashion is one of the main advantages of treatment with S53P4 BAG compared to the traditional treatment with antibiotic-loaded poly-methyl methacrylate (PMMA) beads [9,10]. 

During the treatment of osteomyelitis, typically a cortical window is created to be able to debride the infected bone. The cortical window generates a discontinuity in the cortical bone and together with the debridement, the procedure results in a large bone defect. As osteomyelitis frequently occurs in load-bearing locations like the distal tibia, mechanical stability after treatment should be taken into account. To date, little is known about the mechanical stability of bones with implanted BAG granules. Previously, we have shown that BAG granules, alone or mixed with bone morsels, can withstand the high compressive forces associated with walking in confined compression, with little subsidence [11]. Hence, BAG could potentially reinforce a bone with a large defect. 

In these earlier studies, however, the stiffness of the BAG was measured in a confined compression test. In the clinical situation, the BAG will be only partly confined. Moreover, the bone in which the BAG is placed will have a cortical window, which in fact reduces the strength of the bone. The actual load bearing capacity of a bone treated with BAG will depend on many parameters, among which are the size of the bone and the cortical window in it, the composition of the bone morsels and BAG granules mixture, and the impaction used.

In the present study, we aimed to identify the role of these components in order to better understand and predict the load-bearing capacity of bones treated with BAG. The first objective of this study was to investigate the influence of (cortical) defect geometry on the whole bone mechanical properties. For this purpose, a finite element model representing a bone with a defect was validated first relative to an experimental test and then used to investigate the effect of different defect sizes on the stiffness and strength. A second objective was to investigate if the stiffness of BAG/bone mixtures can be estimated from micro finite element (µFE) models based on micro computed tomography (µCT) images. For this purpose, µFE models were generated from confined compression tests of mixtures of BAG and bone morsels and the material constitutive model parameters were selected to obtain good correspondence between experimental and simulation results. A final purpose was to investigate to what extent these implant mixtures may carry load in a more realistic situation. For this purpose, a high-resolution peripheral quantitative CT (HR-pQCT) scan of the distal tibia of a healthy subject was virtually operated to create a cortical window, and was subsequently virtually reamed and filled with a BAG or bone/BAG implant, to calculate the load sharing between bone and the implant.

The results show that an increased width of a cortical window decreased the bending stiffness of a load-bearing bone significantly more than an increased length. Likewise, estimated failure moments were much more reduced for changes in width compared to changes in length. However, the lost stiffness due to the cortical defects is suggested to be partially restored with the implantation of BAG granules or a mixture of bone morsels and BAG granules. It was observed that the BAG granules carried about 25% of the applied load in a compression simulated on a virtually grafted human tibia.

## 2. Materials and Methods

### 2.1. Effects of Cortical Defect Size and Geometry on Bone Stiffness and Moment of Failure

**Figure 1:**
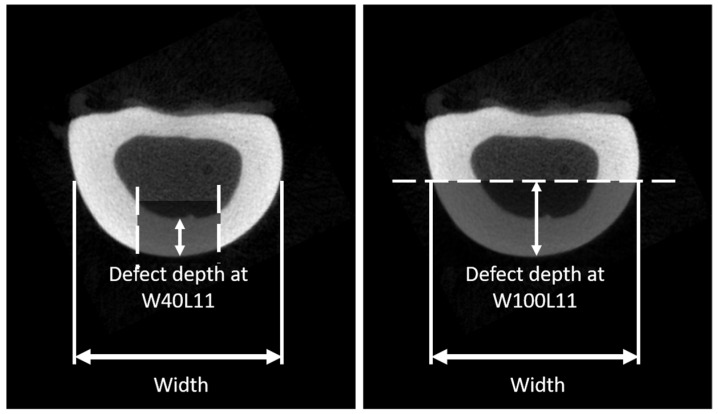
A schematic to define the projected width of the sheep radius and the defect depth, which is directly dependent on the defect width. For the smaller defects, the width is defined as the cortical thickness, while for the wider defects, the thickness is defined as radius in the short axis.

A computational model was developed from µCT images from 10 intact sheep radii (age and sex unknown), obtained from the local slaughterhouse (VION Food Group, Boxtel, the Netherlands). The radii were imaged with µCT (µCT80, Scanco Medical AG, Brüttisellen, Switzerland) using the following settings: Isotropic voxel size 74 µm, energy 70 kVp, intensity 114 µA, and integration time 300 ms. For all radii, a length of approximately 130 mm was scanned. The bone was segmented from the greyscale images using a fixed threshold of 485 mgHA/cm^3^. Subsequently, these segmented voxels were converted into brick elements. Material properties were set to linear elastic with a Young’s modulus of 20 GPa and a Poisson ratio of 0.3 [12,13,14]. For each of the 10 radii, seven differently sized defects were virtually created in the shaft of the radius, with the width specifically adjusted per sample as a percentage of the width (as depicted in Figure 1) of the diaphysis and several fixed heights (Table 1). The sizes of the cortical windows were clinically relevant, as they were based on consultation with an expert in osteomyelitis treatment (author JG: orthopedic surgeon). Boundary conditions prescribed an angular displacement of 0.01 rad at both the distal and proximal bone end, leading to a state of pure bending with the bending axes chosen in such a way that the defect region was compressed. From the angular displacement, the bending stiffness and estimated failure moment were determined, using IPLFE v2.02 (Scanco Medical AG). The bending stiffness was calculated from the resulting reaction moment over the applied rotation. To estimate the failure moments [Nmm], a criterion according to Pistoia et al. was used, which describes failure of the bone when more than 2% of the elements in the bone are strained more than 0.7%; the prescribed Young’s Modulus for the bone in these estimations was 6829 MPa [12,15,16] and the energy equivalent strain was used. The failure moments were estimated for each virtually created defect (Table 1 and Figure 1) and for the intact bone. To determine the reduction in failure moment, the failure moment estimated for a defect was divided by the failure moment estimated for the corresponding intact case (pairwise), resulting in a ratio. The reduction was then defined as 1 minus this ratio.

**Figure 2:**
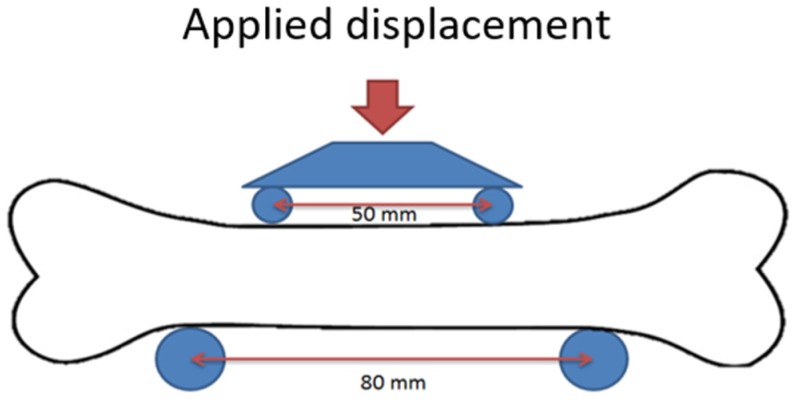
The setup of the four-point bending test. The defect is created at the compressed site of the bone, which was the anterior side of the radius.

To validate the in silico results, non-destructive four-point bending tests were performed for the 10 bones using a tensile test machine (Figure 2) (Zwick Roell Z010, load cell 10 kN, Ulm, Germany). Bones were placed such that the anterior side faced upwards. A preload of 5 N was applied (at 0.5 mm/s), after which the load was increased to 500 N at 0.05 mm/s. Each measurement was performed in triplicate and the stiffness was determined from the last two tests by determination of the slope of the force-displacement graph in the range 400–500 N. The moment was used to evaluate the bending stiffness of the radii. After these experiments with the intact bone, a typical defect (W50L18 in Table 1) with cortical window was created at the anterior side in the middle of the diaphysis of the bone by an orthopedic surgeon. The four-point bending tests were repeated in the same position, such that the defect region was subjected to compression. Again, the bending stiffness was determined and both measured bending stiffnesses were used to validate the prediction of the previously described µFE model, by comparing the final results. As the bones were used also for another study, unfortunately it was not possible to measure their strength.

### 2.2. Determination of Material Properties of BAG/Bone Mixtures

For the present study, we used µCT scans and mechanical data obtained from confined compression tests of BAG/bone mixtures obtained in an earlier study [11]. In summary, bone morsels from seven human femoral heads were milled with a surgical mill to obtain morsels with a size of 3–5 mm. The allograft bone morsels were mixed with bioactive glass granules (2–3.15 mm, Bonalive^®^ Biomaterials Ltd., Turku, Finland), using 5 different ratios: 0, 25, 50, 75, and 100 vol% BAG granules. For each of these groups, 5 samples were tested. They were then subjected to impaction and confined compression, after which they were µCT imaged (isotropic voxel size 36 µm, energy 70 kVp, intensity 114 µA, and integration time 300 ms) [11]. In the current study, these µCT scans were used to derive material properties for the material models. The first image processing step involved an intensity correction, as the BAG granules introduced beam hardening artefacts. For this, the BAG-granule intensity at the center and at the boundary of the image was measured, and a linear intensity correction was applied in the radial direction such that the granules would have the same intensity throughout the image. This correction was dependent on the BAG content and no correction was used for the 0% group. Next, the bone was segmented from the images using a lower and upper threshold of 515 and 838 mgHA/cm^3^, respectively, while the BAG was segmented using a lower threshold of 838 mgHA/cm^3^. In addition, an interface layer around the BAG granules was created, to correct for sliding and contact phenomena at the BAG granules—bone interface that was otherwise not modelled in this continuum model approach. If ignored, this would lead to a much stiffer implant than it would be in reality. To create the interface layer, the segmented BAG granules were eroded by one voxel, followed by a two-voxel dilatation. The interface layer around the BAG granules was then created by subtraction of the eroded image from the dilated image. The final model thus identified three phases: Bone, BAG, and BAG-interface, with the latter overruling the bone or BAG phase.

The segmented bone, BAG, and interface voxels were converted to linear elastic brick elements. Material properties of BAG were based on the literature [17]. Material properties of bone morsels and the BAG interface layer were determined by fitting FE results to experimental measurements. To obtain the material properties for the bone morsels, the samples in the 0% BAG group were used. In this group, no interface layer was needed, as no BAG was incorporated. The Young’s modulus of bone was tuned to make the predicted aggregate modulus equal to that measured in the experimental confined compression tests [11]. Once the material properties for the bone morsels were determined, the 25, 50, 75, and 100 vol% BAG samples were used to determine the material properties of the interface layer. With this approach, material properties of the interface layer are determined by iteratively finding the modulus that best predicts the experimental confined compression modulus for all tested combinations of BAG and bone morsels. Pairwise-comparison (Wilcoxon signed rank) between the experimental and model derived effective moduli was used to evaluate the goodness of the material parameters for the interface layer.

### 2.3. Investigation of BAG-mixture and Bone Load Sharing in the Distal Tibia

**Figure 3:**
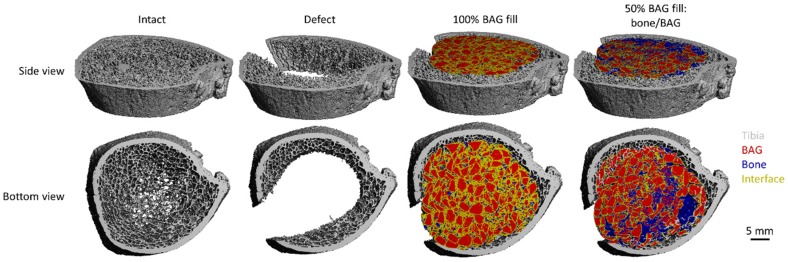
The four conditions of the tibia model viewed from the side and the bottom of the scanned part of the tibia.

To investigate the load sharing in a more realistic clinical case, HR-pQCT images of eight healthy subjects were used (age: 62.1 years ± 13.2; male: 3, female: 5; normal bone mineral index (BMD): 7, osteopenic BMD: 1). These HR-pQCT images were obtained in an observational cohort study in patients with a recent fracture (prospective evaluation of bone strength, physical activity, falls, subsequent fractures, and mortality; approved by medical ethics committee (METC NL45707.072.13), all patients signed informed consent). These images were obtained at VieCuri Medical Center in Venlo with a HR-pQCT system with an isotropic voxel size of 60.7 µm (XtremeCT II, SCANCO Medical AG). The scanning region comprised the default 10.2 mm region located near the distal end of the tibia. The bone was segmented from these images by applying a fixed lower threshold of 387 mgHA/cm^3^, based on the attenuation coefficient histogram. The segmented voxels were then converted into linear elastic brick elements, with a prescribed Young’s modulus of 6826 MPa and a Poisson ratio of 0.3 [16,18]. A “high friction” compression experiment was used to determine the stiffness of the segmented tibia. In the same tibia, a large defect with cortical window was simulated by virtually removing a part of the cortical and part of the trabecular bone from the region of interest prior to the segmentation (a cortical window of approximately W25L10 was simulated). The same high-friction compression (FE) was applied to determine the stiffness of the new situation. A third condition was added by virtually implanting a graft material. The previously described models of the 50 and 100 vol% BAG samples were used (referred to as bone/BAG and BAG, respectively). For this, the µFE models obtained from the confined compression experiment described earlier were scaled to 60.7 µm brick elements, and then used to fill the defect created earlier (Figure 3). This grafted condition was then, again, subjected to the high-friction compression (FE) to determine the stiffness in of the entire bone model. In addition, how the load was divided over the different materials was evaluated to determine the way the load was shared over the different materials.

### 2.4. Statistical Analysis

The open-source software R (version, 3.4.2, packages: Rcmdr) was used to perform all statistics [19,20,21]. Significance was considered for a *p*-value < 0.05.

The effects of cortical defect size and geometry on bone stiffness was determined by a Welch test; because of the assumptions of heteroscedasticity (Levene’s test *p*-value = 0.0466), and a normal distribution (Shapiro–Wilk *p*-value = 0.0969). The statistical differences for the reduction in failure moment were determined by ANOVA, because here homoscedasticity (Levene’s test *p*-value = 0.529) and a normal distribution (Shapiro-Wilk *p*-value = 0.258) could both be assumed. For both the stiffness and the failure moment, post-hoc analysis was performed with a correction for multiple testing (Tukey), taking into account only the differences between the different defects in one sample (repeated measures).

To investigate the stiffness of the BAG-mixture and bone load sharing in the distal tibia, an ANOVA was performed, as homoscedasticity and a normal distribution were assumed (Shapiro–Wilk and Levene’s tests, all *p*-values >> 0.05), again using a repeated measures approach. The goodness of fit of the material properties for bone morsels and the interface layer was determined via a Wilcoxon signed rank test between the experimentally and computationally derived effective moduli. A non-parametric approach was used as the Shapiro–Wilk tests for both groups (experimental and model moduli) resulted in a *p*-value < 0.05 (0.049 and 0.048, respectively). In addition, linear regression was applied on the experimental moduli plotted against the model derived ones.

## 3. Results

### 3.1. Effects of Cortical Defect Size and Geometry on Bone Stiffness, Stress Distribution, and Failure Moment

#### 3.1.1. Bone stiffness

The geometries of the created defects were dependent on the width of the defects as presented in Table 1 and Figure 1. The actual sizes are presented in Table 2.

**Figure 4:**
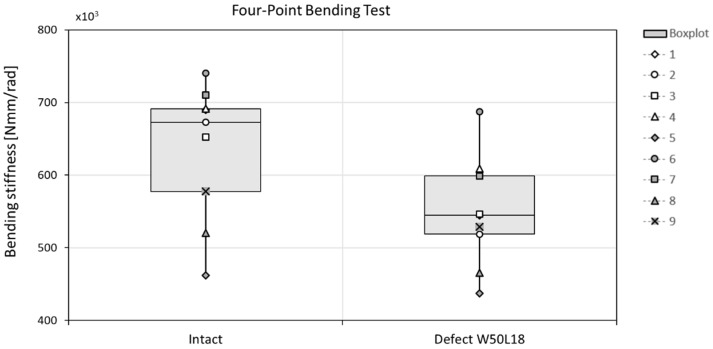
The bending stiffness decreases when a cortical window is created in the center of a sheep radius diaphysis, determined with a four-point bending test. The differences were not significant.

**Figure 5:**
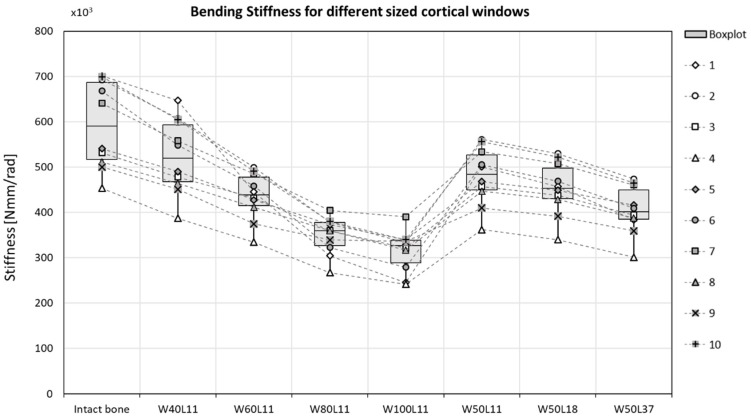
Varying the cortical window size does significantly change the maximum bending stiffness, before failure. The bending stiffness seems to be more affected by a change in width (defects 1–5) than by a change in height (defects 5–7). *p*-values are reported in Table 3.

The bending stiffness was decreased by 13% in sheep radii when a cortical defect (W50L18) was created (Figure 4). This stiffness decrease was overestimated by the µFE model, where an average decrease of 23% was predicted (Figure 5). The bending stiffness predicted for the intact case did not significantly differ from the experimental derived bending stiffness.

In the µFE models, the bending stiffness of the bones was significantly decreased after a defect was created, except for the case with the smallest defect (W40L11) (Figure 5 and Table 3). Increasing the width of the gap by more than 20% led to a significant reduction in stiffness. Increasing the length while keeping the width constant, however, did not significantly decrease the stiffness. The average bone stiffness that was maintained after the differently sized cortical windows were created are specified in Table 4. Increasing the width of the defect with 10% led to a decrease in bending stiffness of approximately 7%. This reduction is similar to the reduction in stiffness observed for a defect that was doubled in length, as increasing it from 18.5 mm to 37 mm showed a reduction of approximately 8% of the total bone bending stiffness (Table 4). In the most severe cases (W80L11 and W100L11), the stiffness was reduced to 59.8 ± 9.4% and 54.0 ± 10.4% of the initial stiffness, respectively. In the case with the longest defect (W50L37), the stiffness was reduced to 68.8 ± 6.9% of the initial stiffness.

#### 3.1.2. Stress Distribution

**Figure 6:**
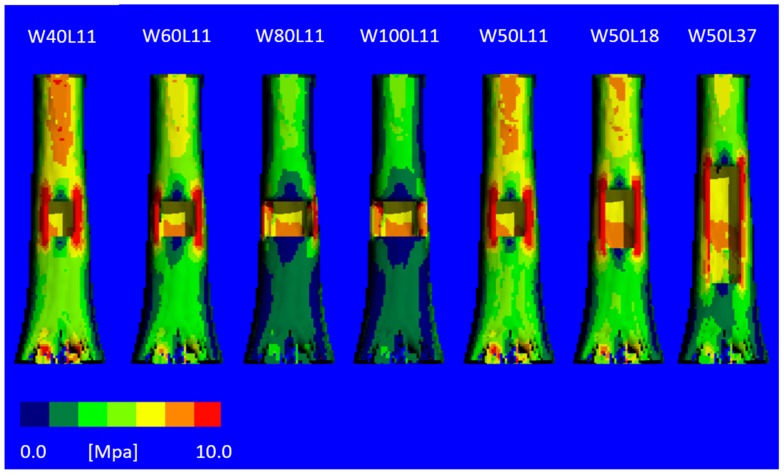
The von Mises stress distribution for virtually created cortical defects with varying sizes, for one of the bone samples. With an increasing width of the defect, the high stresses are more concentrated in the corners of the defects. Defects 3 and 4 show low stresses below the defect and high stresses in small areas, whereas the narrow defects show a more homogeneous distribution of the stresses.

The von Mises stress distribution (Figure 6) revealed severe unloading of the bone at the anterior side where the cortical window is created with increasing width, explaining the reduced stiffness. For the models with an increased window length, this effect is less severe.

#### 3.1.3. Failure Moment

**Figure 7:**
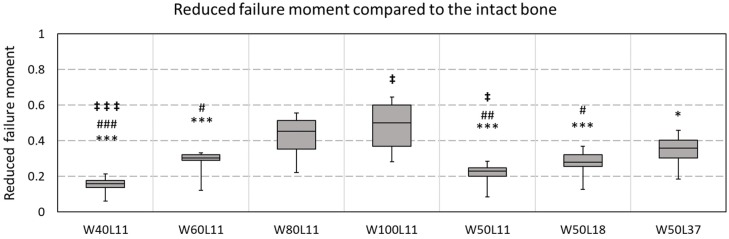
The reduced failure moment due to the defect geometry. The reduction in failure is defined as $\left(1-\frac{failure\;moment\;estimate\;defect\;case\;\left[Nmm\right]}{failure\;moment\;intact\;bone\;\left[Nmm\right]}\right)$. A pairwise ANOVA (with Tukey correction) showed significant differences between the reduction; * indicates a difference with W100L11, # indicates a significant difference with W80L11, ‡ indicates a difference with W50L37. Triple signs indicate a *p*-value < 0.001, double signs a *p*-value < 0.01, and single signs a *p*-value < 0.05.

Failure moments were estimated for all virtually created defects and compared to the corresponding intact bone (Figure 7). For the smallest tested defect (W40L11), a reduction in failure moment of 15 ± 5% was observed. Doubling the width while keeping the length constant (W80L11) resulted in a significantly different reduction of 42 ± 12%. For the widest tested defect (W100L11), a reduction of 48 ± 14% of the initial failure moment was observed. This was significantly different from reduction observed for the longest defect (W50L37) tested, which was 35 ± 10%. This was, on the contrary, not significantly different compared to the situation with the defect that was half this length (W50L18), which showed a reduction of 27 ± 9%. 

### 3.2. Determination of Material Properties of BAG/Bone Mixtures

**Figure 8:**
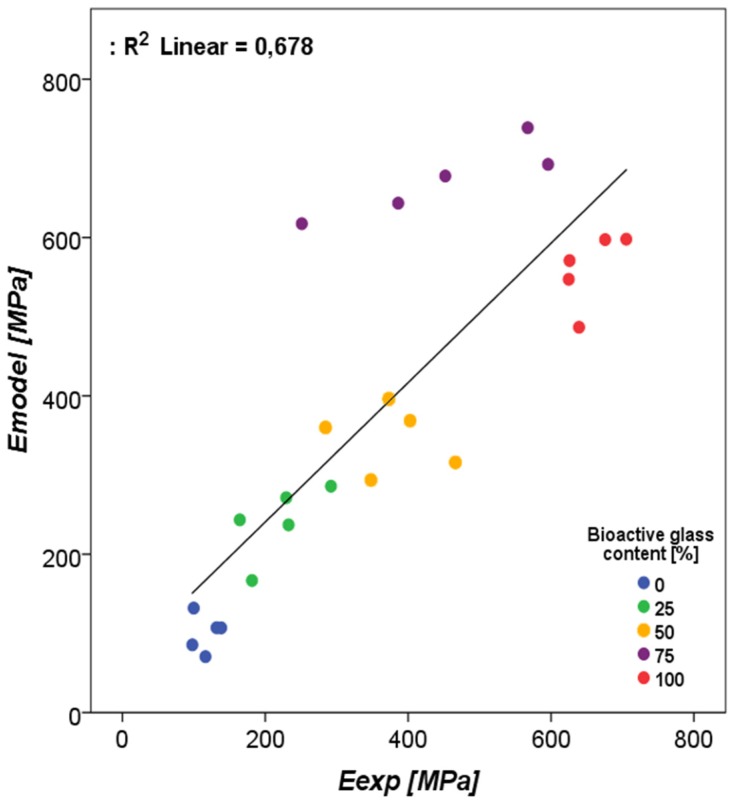
The regression of the experimental compression moduli as measured by Hulsen et al. (2016) [11] vs. the estimated compression moduli for the materials with increasing bioactive glass contents.

The fitting of the aggregate moduli for the 0% BAG group resulted in a bone morsel Young’s modulus of 2.5 GPa (Table 5). For the interface layer, the best agreement between calculated and measured aggregate moduli was found for a Young’s modulus of 25 MPa. The pairwise comparison between the experimentally derived and the model-derived effective moduli resulted in a *p*-value of 0.77. The R^2^ obtained with regression was 0.678 (Figure 8). The material model that was deviating most was the 75% bone/BAG group.

### 3.3. Investigation of BAG-Mixture and Bone Load Sharing in the Distal Tibia

**Figure 9:**
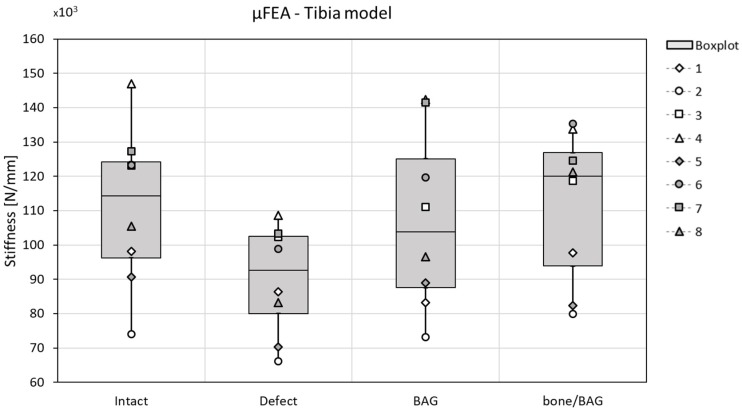
The stiffness related to the condition (x-axes) in the µFE tibia model; the stiffness decreases when the defect was created in all cases, and generally increased again with grafting (both for bioactive glass (BAG) and bone/BAG). The differences were not statistically significant.

As expected, the stiffness decreased when the defect is created and increases again when grafted with BAG or a mixture of 50% bone morsels and 50% BAG (bone/BAG). None of the changes, however, reached the level of significance, and no specific differences between the two graft materials were observed (Figure 9). Some small differences are observed in the load division over the different materials (tibia bone, BAG, and bone morsels) at the distal and proximal side of the tibia. When the defect was grafted with BAG, distally, 25.8 ± 3.7% of the load was carried by the BAG and 73.0 ± 3.9% by the tibia bone. Proximally, this was 22.6 ± 3.8% and 75.3 ± 3.9%, respectively. When the defect was grafted with the bone/BAG mixture, the BAG carried 28.5 ± 5.6%, the tibia bone 64.9 ± 4.5%, and the bone morsels 9.7 ± 1.8% of the load at the distal site. Proximally, the BAG granules carried 32.1 ± 6.0%, the tibia bone carried 63.3 ± 8.1%, and the bone morsels carried 6.3 ± 0.8% of the total applied load in compression. In both implantation groups, the load carried by the interface layer at the proximal/distal surfaces was <0.5%.

## 4. Discussion

In the treatment of chronic osteomyelitis, a large defect with a cortical window needs to be created. In many cases, the osteomyelitis is located in a load-bearing site, of which the mechanical stability will be reduced by the creation of such a large defect with cortical window [8,22]. Therefore, the first objective of this study was to assess how the size of the cortical window influences the bending stiffness and stress distribution in a load-bearing bone. The results of a computational model show that the size of the cortical window does significantly influence the bending stiffness of the bone. With an increasing width of the defect, the bending stiffness significantly decreases (Figure 5 and Table 3). As expected from beam theory, this effect is less substantial, and not statistically significant, for an increase in length of the defect [23]. The two widest simulated cortical windows (W80L11 and W100L11) resulted in average bending stiffnesses that were only 59% and 53% of the initial bending stiffness, while the average bone bending stiffness calculated for the longest defect (W50L37) was still 68% of the initial bending stiffness. The bending stiffness determined with the FE model were similar to the results obtained with the four-point bending tests for the intact bones, but overpredicted the loss in stiffness for the cases with defect.

Besides the bending stiffness, the failure moment was estimated for the intact bones and all defect cases. This moment was estimated with making use of the criterion according to Pistoia et al. [15,16], which has not been validated for this loading mode. Although the accuracy of this strength prediction thus is not known, it is expected to provide reasonable strength comparisons between cases as investigated here. The obtained failure moment estimates confirmed the observed effects in stiffness, as an increase in width substantially reduces the failure moment more than an increase in the length of a defect. When the width of the defect would be doubled while the length was not changed (e.g., W40L11 to W80L11), an additional 27% decrease in failure moment was observed. When the length was doubled with a fixed width (W50L18 to W50L37), only an additional 8% decrease in failure moment was observed. The knowledge gained with these FE analyses on bending stiffness and failure moment is clinically relevant, not only in the treatment of osteomyelitis, but in any situation where a (large defect with) cortical window needs to be created in a load-bearing bone (e.g., in bone tumor removal). 

The second objective of the current study was to investigate if the stiffness of BAG/bone mixtures can be estimated from µFE models based on µCT images. Material models were determined for BAG, bone morsels, and an interface, such that the effective modulus determined with simulated compression results corresponded to the experimentally obtained moduli [11]. The final objective was to investigate to what extent these implant mixtures may carry load in a more realistic situation. Such approach will help to better understand the mechanical interactions between the bone and implant material [24,25]. To evaluate the load-bearing of BAG implants in the current study, the mixtures scanned in the earlier experiment were virtually implanted in human tibiae to evaluate the effect on the stiffness and load-transfer in the bones. The stiffness found for the intact bones was found in the expected kN/mm range, although slightly lower than previously reported with similar HR-pQCT-based predictions [26]. As the resolution of the scans of the earlier experiment was higher than that of the human scans, the mixture model voxels were upscaled in size. Although this scaling will increase the size of the grains, morsels, and interface layer, it will not affect the (homogenized) modulus of the mixture itself. The interface layer was modelled in our continuum model approach in order to correct for the sliding and contact phenomena at the BAG granule boundaries. Although this is a simplification, ignoring it would have led to a much stiffer implant than it is in reality. Although not statistically significant, our patient-specific predictions suggest that the stiffness lost after creating the cortical window can be restored by the implantation of a mixture of bone morsels and BAG granules or by BAG granules alone. In both simulated implantation groups, the BAG granules carried a substantial amount (around 25%) of the total applied load. This way, the granules were able to support the defect tibia bone in a load-bearing application. A combination of BAG and bone morsels carried more load than BAG alone, and thus further reduced the load carried by the tibia bone, however this difference was not significant. The virtual creation of the cortical window and the virtual implantation of both the BAG and bone/BAG in these models was performed manually. It should be noted, though, that this modelled case is an idealized case, that maximizes load transfer through the implanted material due to the confined conditions at the proximal and distal end and the prescribed displacement boundary conditions applied. The numbers found here should thus be considered a best-case scenario.

To prevent the results from being influenced by differences between subjects (e.g., in size, sex, age, or BMD), a pairwise comparison approach was used in both sheep radii experiments, as well as the human tibiae experiments. This means that results from the same subject were statistically compared in the different conditions (intact, defect(s), and grafted). This will eliminate the influence of subject specific factors and will result in a more solid final result.

An important limitation of this study is that the stiffness restoration observed here is only valid initially after implantation of the BAG. The interface layer was only modelled, tuned, and validated against experimental results in the case of loose granules. Over time, BAG will bond to bone and degrades, which will change the mechanical properties of the mixture [2,3,27]. To evaluate to what extent the mechanical properties are affected over time, it would be needed to monitor the stiffness of bone with BAG mixtures during bone ingrowth. Obviously, this would be possible only in an animal experiment. This should be considered for future research as, to date, hardly any literature is available about the in vivo mechanical behavior of BAG materials. 

In conclusion, bone grafts/BAG mixtures can potentially unload the cortical bone that is weakened by a large defect directly after the operation by up to approximately 25%, but only in the case of optimal load transfer through the mixture. 

## Figures and Tables

**Table 1:** Different cortical window sizes to test the effect of defect size on the stress distribution. The width of the radius is defined as projection width of the diaphysis as indicated in Figure 1.

Defect	Defect Width (Circumferential Direction)	Defect Length (Longitudinal Direction)
W40L11	40% of width	11.1 mm
W60L11	60% of width	11.1 mm
W80L11	80% of width	11.1 mm
W100L11	100% of width	11.1 mm
W50L11	50% of width	11.1 mm
W50L18	50% of width	18.5 mm
W50L37	50% of width	37.0 mm

**Table 2:** Defect depth, width, and area in absolute values as mean and standard deviations.

Measure	W40L11	W60L11	W80L11	W100L11	W50L11	W50L18	W50L37
Width (SD) (mm)	0.72 (0.042)	0.90 (0.053)	1.08 (0.063)	1.44 (0.085)	1.81 (0.106)	0.90 (0.053)	0.90 (0.053)
Defect area (SD) (mm^2^)	8.01 (0.469)	10.02 (0.587)	12.02 (0.704)	16.03 (0.939)	20.04 (1.174)	16.70 (0.978)	33.40 (1.957)
Defect depth (mm)	3.3 (0.27)	3.3(0.27)	3.4 (0.43)	4.1 (1.35)	6.0 (0.99)	3.3 (0.27)	3.3 (0.27)

**Table 3:** Pairwise comparison (Welch’s test with Tukey correction) of the bending stiffness of bones with varying cortical defects leads to many statistical differences: ^a^ *p*-value < 0.001 and ^b^ *p*-value < 0.05.

Defect Size	Intact	W40L11	W60L11	W80L11	W100L11	W50L11	W50L18	W50L37
Intact	x							
W40L11	0.23368	x						
W60L11	<0.001 ^a^	0.05865	x					
W80L11	<0.001 ^a^	<0.001 ^a^	0.06319	x				
W100L11	<0.001 ^a^	<0.001 ^a^	0.00160 ^b^	0.92728	x			
W50L11	0.00395 ^b^	0.79351	0.78240	<0.001 ^a^	<0.001 ^a^	x		
W50L18	<0.001 ^a^	0.22967	0.99873	0.01159 ^b^	<0.001 ^a^	0.98115	x	
W50L37	<0.001 ^a^	0.00232 ^b^	0.96104	0.51423	0.04416 ^b^	0.16915	0.70436	x

**Table 4:** The reduced bone bending stiffness due to the cortical window geometries.

Defect	Maintained Part of Initial Bending Stiffness (%)
W40L11	88.3 ± 3.1
W60L11	74.0 ± 5.6
W80L11	59.8 ± 9.4
W100L11	54.0 ± 10.4
W50L11	81.3 ± 5
W50L18	76.8 ± 5.9
W50L37	68.8 ± 6.9

**Table 5:** Prescribed material properties for each phase in the human tibia high-friction compression (FE) model.

Phase	Young’s Modulus (MPa)	Poisson Ratio (-)
Bone morsels	2.5 × 10^3^	0.3
BAG	35 × 10^3^	0.3
Interface	25	0.3
Tibia bone	6826	0.3
